# A high-throughput, restriction-free cloning and screening strategy based on *ccd*B-gene replacement

**DOI:** 10.1186/1475-2859-13-38

**Published:** 2014-03-10

**Authors:** Bjarte Aarmo Lund, Hanna-Kirsti Schrøder Leiros, Gro Elin Kjæreng Bjerga

**Affiliations:** 1 NorStruct, Department of Chemistry, Faculty of Science and Technology, UiT - The Arctic University of Norway, N-9037 Tromsø, Norway; 2 Uni Research AS, Centre for Applied Biotechnology, High Technology Centre, Thormøhlensgt. 55, N-5008 Bergen, Norway

**Keywords:** Restriction-free cloning, Exponential megapriming PCR, High-throughput, Counterselection, Recombinant DNA technology, Parallel cloning

## Abstract

**Background:**

In high-throughput demanding fields, such as biotechnology and structural biology, molecular cloning is an essential tool in obtaining high yields of recombinant protein. Here, we address recently developed restriction-free methods in cloning, and present a more cost-efficient protocol that has been optimized to improve both cloning and clone screening.

**Results:**

In our case study, three homologous β-lactamase genes were successfully cloned using these restriction-free protocols. To clone the genes, we chose a gene replacement strategy, where the recombinant genes contained overhangs that targeted a region of the expression vector including a cytotoxin-encoding *ccd*B-gene.

**Conclusion:**

We provide further evidence that gene replacement can be applied with high-throughput cloning protocols. Targeting a replacement of the *ccd*B*-*gene was found to be very successful for counterselection using these protocols. This eliminated the need for treatment with the restriction enzyme *Dpn*I that has so far been the preferred clone selection approach. We thus present an optimized cloning protocol using a restriction-free *ccd*B-gene replacement strategy, which allows for parallel cloning at a high-throughput level.

## Background

To meet the demands in biotechnology and structural biology, high-throughput molecular cloning methods have been developed to obtaining sufficient amounts of recombinant protein in a parallel manner. For this purpose, restriction-free (RF) cloning is a simple PCR-based approach for inserting any DNA fragment into any position of a vector, independently of restriction-sites, ligation, without elaborate pretreatments of the vector and without sequence constraints [1-3]. Today’s high fidelity polymerases used for cloning have extraordinary low error rates. Therefore, PCR amplification of large DNA regions, such as entire plasmids, is no longer challenging. The RF-method relies on site-specific insertion of the gene of interest to the vector by linear plasmid amplification [2,3]. Briefly, the insertion requires that the gene of interest is flanked by sequences complimentary to the insertion site of the vector. These overhang regions are generated during a standard PCR amplification from the relevant template using primers containing both a gene-specific region and an adapter region. Finally, the amplified gene with overhangs is used as a megaprimer in the second PCR, where the insertion takes place during linear plasmid amplification. The complementary DNA products form a plasmid containing two single-stranded nicks, which is ready for transformation and clone screening. To shorten the protocol, the gene-specific and the linear plasmid amplification can be combined in a single PCR reaction. Exponential megapriming PCR (EMP) cloning addresses the major shortcoming of the published RF-cloning protocol, the product- and size-limiting linear plasmid amplification, by introducing a reverse vector-specific primer making the amplification exponential [4]. This protocol, however, requires two additional, successive steps of phosphorylation and ligation to close the plasmid. The fact that these cloning regimes requires absolutely no pretreatments of the vector, is in great contrast to other high-throughput cloning methods, such as TA-cloning and ligation-independent cloning [5-7]. This makes these protocols beneficial for many high-throughput applications, such as structural genomics and biotechnology.

The RF- and EMP-protocols rely on a screening process to select positive clones from background clones. This screening includes treating the product mix from the second PCR with the restriction enzyme *Dpn*I [8]. The enzyme will remove the methylated parental DNA before transformation, such as established for the mutagenesis protocol [9,10]. Typically, *Dpn*I is incubated with the substrate at 37°C for an hour. The efficiency is improved by longer incubations, up to several hours, with the risk of over-digestion. Also, efficiency of *Dpn*I is dependent on buffer and the methylation state of the template DNA, and requires vector propagation in Dam^+^ strains [11].

In our study, we have tested an alternative selection approach using the coupled cell division B gene *(ccd*B) gene, that has previously been reported for recombinational cloning and, more recently, the sequence and ligation independent cloning (SLIC) and fragment exchange (FX) cloning [12-16]. The negative selection is based on the presence of the *ccd*B*-*gene in the cloning region of the vector. In recombinant DNA the toxic gene becomes replaced with the gene of interest. In negative clones, however, a lethal protein is encoded from the *ccd*B-gene that interferes with the DNA gyrase activity and cause un-repairable chromosomal damage to the cell and ultimately cell death [17,18].

In our case study, we have investigated using a gene replacement principle [2] in combination with negative *ccd*B selection to improve the clone screening process of RF- and EMP-cloning. In this study, three homologous *bla*_OXA_*-*genes, encoding the oxacillinase (OXA) β-lactamases OXA-48, −181 and −245 [19-21], were successfully cloned downstream of a T7 promoter. We carried out the cloning by replacing a 1.7 kb region of the Gateway® vector, pDEST17, which is designed for selection based on the *ccd*B-gene, with our genes of interest, not by traditional recombination, but by using the newly invented RF- and EMP-cloning protocols. We show that RF- and EMP-cloning protocols can successfully be used in gene-replacement applications. Such applications increase the potential of this method in the manipulations of plasmids. Also, we have found that *ccd*B*-*selection was a successful approach for clone selection for the newly invented cloning regime, which eliminates the need for *Dpn*I removal of parental DNA. Taken together, we present an improved, cost-efficient and less labour-intensive cloning protocol, which allows for cloning and screening in a parallel fashion, using *ccd*B-gene replacement. Our data increase the potential of the cloning method in high-throughput technologies.

## Results and discussion

RF-cloning, including the optimized EMP-protocol, is a simple method for inserting any gene into a vector, independently of restriction-sites [2-4]. The benefit of the method is that is has no requirements for pretreatments of vector. The method holds a great potential in biotechnology, structural biology and other high-throughput demanding fields.

**Figure 1:**
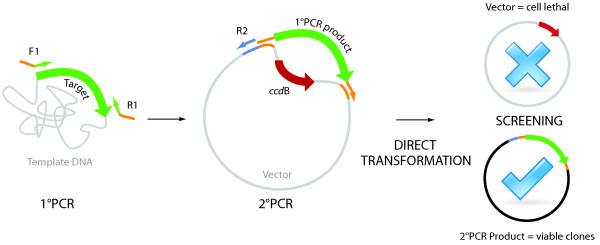
**Principle of *ccd*B-gene replacement strategy.** The schematic representation of the *ccd*B-gene replacement strategy follows three simple steps, two PCR reactions and a direct transformation of the second PCR product. Primers F1 and R1 are used in a gene-specific amplification in the first PCR round. Gene-specific regions of primers are indicated by thin, green arrows, whereas the introduced overhang regions are coloured in orange. In the second PCR, the product from the first PCR is used as a megaprimer in linear plasmid amplification. The overhang regions of the product bind to the desired insertion sites in the vector that flank the *ccd*B gene. *ccd*B-gene for negative selection becomes replaced during amplification of the plasmid. The R2 primer can be used for exponential amplification. Complementary primer-binding regions in the vector are marked in the same colours as primers. Finally, the parental vector and the product from the second PCR can be transformed directly to competent cells. Those cells that take up the paternal vector will be subjected to un-repairable chromosomal damage caused by a toxin encoded by the *ccd*B-gene, and will ultimately die. Positive clones, however, will survive since the negative selection marker gene has been replaced with the gene of interest. Generally, input DNA, such as genomic DNA and vector, is coloured in grey to differentiate it from the black-coloured product DNA. Thin and fat arrows differentiate primers and open reading frames, respectively.

**Figure 2:**
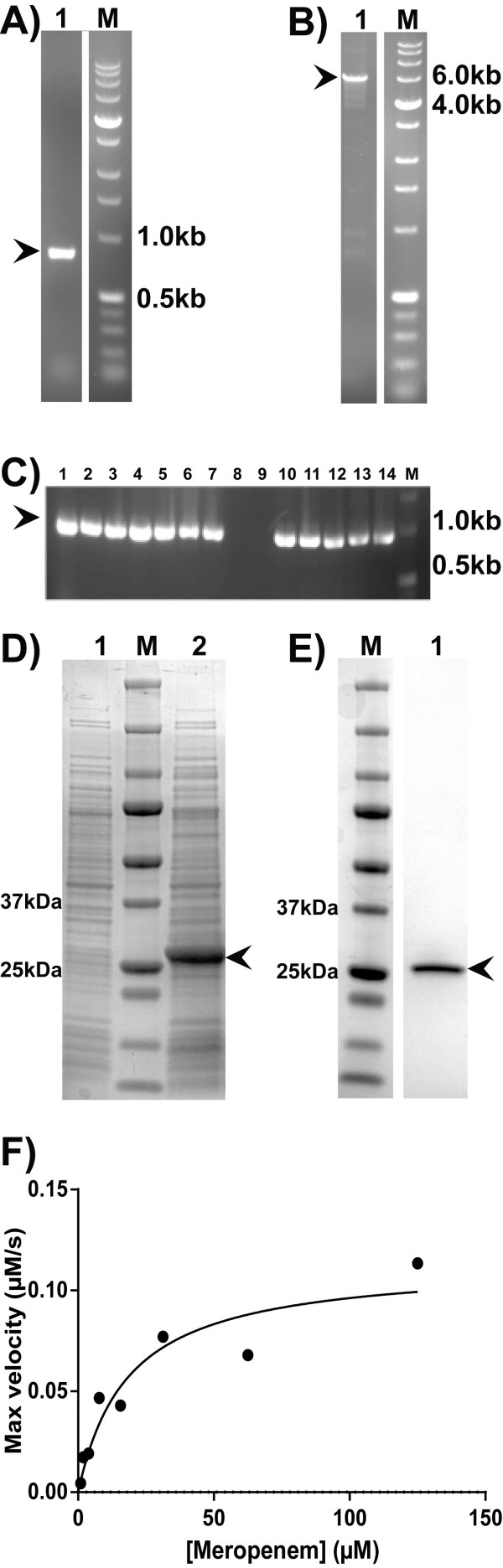
***ccd*B-gene replacement is an efficient cloning strategy in obtaining active, recombinant OXA-protein. A)** Lane 1 shows the PCR products from *bla*_OXA-181_ gene amplification analysed by agarose gel electrophoresis. M, Perfect DNA 1 KB ladder; relevant bands are indicated to the right. Arrow indicates the product matching the expected size, 857 bp. **B)** Lane 1 shows the PCR products from the *bla*_OXA-181_ gene inserted into pDEST17 by exponential plasmid amplification and analysed on agarose gel. M, Perfect DNA 1 KB ladder; relevant bands are indicated to the right. Arrow indicates the plasmid product matching the expected size, 5408 bp. **C)** Lane 1–14 shows the results from colony PCR analysis of clones 1–14. The expected size of the screened inserts is 948 bp, and arrow indicates the position of products matching the expected size. **D)** SDS-PAGE analysis of the expression of recombinant OXA-48. Lane 1 shows the uninduced control, whereas lane 2 shows the IPTG-induced recombinant OXA-48 protein expressed in *E. coli* strain BL21Star(DE3)pRARE. M, Precision Plus protein standard; relevant bands are indicated to the right. Arrow indicates the position of induced protein matching the theoretical mass of OXA-48 with the leader sequence removed (28 kDa). **E)** Recombinant OXA-48 protein was isolated from the periplasm, and purified through two anionic exchange steps. The integrity of the purified protein is shown in lane 1. M, Precision Plus protein standard; relevant bands are indicated to the right. **F)** The β-lactam antibiotic, meropenem, is hydrolysed by OXA-48. The hydrolysis velocities (μM/s) were plotted as a function of Meropenem concentration (μM); the enzyme follows the Michaelis-Menten kinetics. Based on these data, *K*_m_ was calculated to be 18 ± 5 μM and *k*_cat_ to 0.23 ± 0.02 s^−1^, giving a catalytic efficiency of 1.2 × 10^4^ (M^−1^ s^−1^).

Using these restriction-free cloning protocols we inserted the desired 798 bp long, homologous genes, *bla*_OXA-48_, *bla*_OXA-181_ and *bla*_OXA-245_, downstream of the T7 promoter by replacing a region of the commercial vector pDEST17, including the *ccd*B-gene (principle outlined in Figure 1). The genes of interest were amplified in a standard PCR reaction from a genomic DNA template isolated from clinical isolates (Figure 2A). Primers contained both a gene-specific sequence, which determined the amplification of the entire reading frame of the gene, and an adapter region complementary to the insertion site of the vector. Those two insertion sites were 1744 bp apart in the vector; the spanned region included the *Cam*R-*ccd*B cassette. The amplified genes containing adapter regions were used as megaprimers in a second PCR, where the insertion and replacement reaction took place during linear plasmid amplification (Figure 2B). Although the efficiency of the amplifications is generally high due to the high fidelity polymerases, the product amounts generated in the second PCR can sometimes be low. We assume that product formation could be reduces due to particularly long or difficult GC-rich sequence amplifications or due to the formation of secondary products. This has not been a subject for investigation in this study, but optimization with megaprimer and vector concentrations and thermal cycling conditions may improve product formation. It may be worth noticing that although products cannot be observed after separation on a standard gel electrophoresis, a small amount of product positive clones may still be generated, as commonly known from site-directed mutagenesis protocols. To compensate for low product formation and low colony numbers, exponential plasmid amplification was applied by introducing a reverse primer complimentary to the region upstream to the 5′ insertion site. While the principle of gene replacement has been described [2], the replacement of such a large DNA fragment, constituting almost 30% of the vector, has not previously been reported. Our data increases the potential of RF- and EMP-cloning protocols in the generation and manipulations of plasmids.

In the process of selecting positive clones, *Dpn*I treatment is used for the removal of template DNA in the published RF- and EMP-cloning protocols [2-4]. This procedure is, however, time-consuming and may lead to over-digestion of positive clones. Furthermore, efficiency of *Dpn*I depends on buffer composition and the methylation state of the template DNA, and requires that template DNA has been propagated in Dam^+^ strains. In this report, we have investigated using the gene replacement principle [2] in combination with negative *ccd*B selection [12-15] to improve the clone screening process of RF- and EMP-cloning protocols (Figure 1). The product generated in the second PCR during plasmid amplification can thus be transformed directly to competent cells (Figure 1) without the need of *Dpn*I pre-treatment. Positive clones were identified by standard colony PCR screening using vector-specific primers flanking the insertion site (Figure 2C), and confirmed by Sanger sequencing using the same primers. Generally, cloning was successful independently of linear or exponential amplification, judged from the ratio of positive clones to total screened clones identified by colony PCR (Table 1). Furthermore, induction of recombinant OXA-proteins for heterologous expression in the *E. coli* BL21Star (DE3) pRARE was confirmed; here, recombinant OXA-48 is given as an example (Figure 2D). Isolation from periplasm together with two purification steps yielded a pure, recombinant protein as determined by SDS-PAGE analysis (Figure 2E). The recombinant enzyme displayed activity towards the β-lactam antibiotic meropenem. Data were fitted to the Michaelis-Menten equation; *K*_m_ was calculated to be 18 ± 5 μM and *k*_cat_ to 0.23 ± 0.02 s^−1^, giving a *k*_cat_/ *K*_m_ of 1.2 × 10^4^ (M^−1^ s^−1^) (Figure 2F). These results are within the same range as previously reported [22]. Together with sequencing and expression results, activity data confirm that the gene replacement strategy was successful for obtaining recombinant clones of *bla*_OXA-48._

**Table 1:** **Numbers for *ccd*** **B counterselection with or without *Dpn*** **I for the targets and cloning protocols used**

**Gene target**	**Cloning technology**	***Dpn*** **I treatment**	**Positive/Total screened clones**	**Transformants**^**1**^
*bla*_OXA-48_	RF-cloning	-	13/14	n.d.
*bla*_OXA-245_	RF-cloning	-	12/14	n.d.
*bla*_OXA-48_	EMP-cloning	-	26/28	1550
*bla*_OXA-48_	EMP-cloning	+	27/28	800
*bla*_OXA-181_	EMP-cloning	-	13/29	32
*bla*_OXA-181_	EMP-cloning	+	11/24	24

^1^n.d., not determined.

The replaced 1.7 kb region of the pDEST17 vector containing the *ccd*B-gene is designed to increase the efficiency of clone selection in recombinational cloning [15]. We explored the vectors containing the *ccd*B-gene for selection using other cloning methods more applicable for high-throughput cloning, such as the recently developed RF- and EMP-cloning protocols. These cloning protocols have not, previously, been reported in combination with negative *ccd*B selection. We have also examined *ccd*B-selection in combination with *Dpn*I restriction using the EMP cloning protocols. Parental pDEST17-vectors with frame shifts within the *ccd*B-gene that would not kill the cell would thereby be removed. As an example, when the *bla*_OXA-181_ gene was inserted to pDEST17 by our *ccd*B-gene replacement strategy using the EMP-cloning protocol, thirteen of twenty-nine colonies were confirmed positive by colony PCR screening using vector-specific primers flanking the insertion site (Figure 2C, Table 1). Background clone numbers did not dramatically improve by additional *Dpn*I treatment. Although the success of *ccd*B selection has been reported before [15], we could confirm that the *ccd*B counterselection itself was sufficient for positive clone selection. Similar results were found when we cloned homologous *bla*_OXA-48_-genes using the EMP-cloning protocol (Table 1). Judging from the number of positive clones to total number of clones tested some clones seem to fail in the insert replacement, but still escape the *ccd*B-determined toxicity as determined by negative PCR colony screen. This can be explained by primer-dimer products from the first PCR being inserted to the vector in the second PCR, or simply due to failed colony inoculation to PCR screening reaction. In our study, primer-dimer formations may have been relevant for the gene-specific amplification of *bla*_OXA-181_, leading to false positives in the plasmid amplification and thus a low number of positive clones to total screened clones. Optimizations, such as adding dimethyl sulfoxide (DMSO), could be carried out to reduce the primer-dimer formation during the gene-specific amplification. In cases where *Dpn*I treatment was not performed, frameshifts or deletion of the *ccd*B gene may also have caused false positives.

In one EMP-cloning experiment, eight hundred colonies were obtained for the *Dpn*I-treated sample, whereas more than fifteen hundred colonies were obtained for the untreated sample (Table 1). When selectivity was determined by colony PCR, the *Dpn*I treated sample did not give higher score for positive clone selection. Hence, we hypothesize that *Dpn*I treatment may lead to over-digestion of DNA, thereby reducing the probability of positive clones. This hypothesis was, however, not further investigated.

We chose to explore the commercially available Gateway® vectors encoding a *ccd*B-gene. For labs that have already implemented recombinational cloning, our optimized protocol allows for parallel cloning of PCR products into Gateway® expression vectors harbouring different fusion-tags based on conserved regions shared among the vectors. Other vector series encoding the *ccd*B-gene are, however, academically available [such as described in [12,16,23].

## Conclusions

In our report, we have provided further evidence that gene replacement can be applied with restriction-free cloning protocols, thus, increasing the potential of these methods in the manipulations of plasmids. Also, we have found that *ccd*B*-*selection was a successful method for clone selection for the newly invented cloning regimes. Our optimized procedure represents a more efficient, cost reducing and less labour-intensive approach for molecular cloning and clone screening. Together with previous reports, our data confirms and improves the potential of the RF- and EMP-cloning protocols in high-throughput technologies.

## Methods

### Cloning

Primers were designed to replace the *ccd*B-gene with the *bla*_OXA-48_-genes encoding OXA β-lactamases, OXA-48, −181 and −245 [GenBank: KC757416, JN205800 and JX43800, respectively]. The RF-cloning.org webserver [24] was used to design primers F1 (5′-ATAATTTTGTTTAACTTTAAGAAGGAGATATACAT*ATGCGTGTATTAGCCTTATCGG*, where italic represents *bla*_OXA_ gene-specific region) and R1 (5′-GGCTTTGTTAGCAGCCTCGAATCA*CTAGGGAATAATTTTTTCCTGTTTGAG*) complementary to both inserts and vector. Desalted primers were synthesized by Sigma-Aldrich.

The *bla*_OXA_-megaprimers were amplified in 1x HF Phusion buffer (NEB) using 200 ng genomic DNA from clinical isolates, 200 μM dNTP (VWR), 0.5 μM of both primers F1 and R1, and 1U Phusion DNA polymerase (NEB) in 50 μL final volume. The samples were denatured by heating to 98°C for 30s, followed by 25 cycles of 8s denaturation at 98°C, 20s annealing at 56°C and 15s elongation at 72°C, and the reaction was terminated by a final 5 minutes elongation step at 72°C. PCR products were purified by the Nucleo-Spin gel and PCR cleanup kit (Macherey-Nagel). PCR products were analyzed by gel electrophoresis (1% agarose).

RF-cloning was performed as described in previous reports [2,3]. The linear amplifications took place in 1x HF Phusion buffer, using 100 ng megaprimer, 200 μM dNTP, 1% DMSO (NEB), 25 ng Gateway® pDEST17 vector (Invitrogen) and 1U Phusion DNA polymerase. The mixture was heated to 98°C for 30s denaturation, followed by 30 cycles of 8s denaturation at 98°C, 20s annealing at 54°C and 107s elongation at 72°C, terminated by a final 5 minutes elongation step.

Our EMP protocol was performed as described previously [4], with minor modifications. Exponential amplifications of the plasmids were performed with 25 ng megaprimer, and 1x HF Phusion buffer, 200 μM of dNTP, 0.5 mM of each primer F1 and R2, 25 ng pDEST17 and 1U Phusion DNA polymerase. Both the F1- and R1-primers described above carry overhangs complementary to the insertion site of the vector, in contrast to the reported EMP-cloning, where F1-primer is designed without an overhang [4]. Consequently, the R2-primer (5′-TTCTAGAGGGAAACCGTTGTGGTCT) was designed complementary to the vector region 5′ to the F1-primer to ensure an exponential amplification.

The samples were denatured at 98°C for 30s, followed by 25 cycles of 10s denaturation at 98°C, 30s annealing at 56°C and 107s elongation at 72°C.

The EMP products were first purified as described above, then phosphorylated and ligated by incubating 16.5 μL PCR-product in 1.7 μL 10xT4 DNA ligase buffer (NEB) with 5U T4 polynucleotide kinase (NEB) for 30 minutes at 37°C, followed by the addition of 6U T4 DNA ligase (NEB) and overnight incubation at 4°C.

For *Dpn*I treatment, 20U enzyme (NEB) was added directly to half of the PCR product and incubated at 37°C for 30 minutes. 2 μL reaction products, *Dpn*I treated or not, was then transformed by a conventional heat-shock protocol to competent *E. coli* XL1Blue cells (Stratagene). The mix of transformed bacteria was spread on an LB-agar plate supplemented with 100 μg/ml ampicillin (Sigma). Screening was performed by colony PCR using the vector-specific T7 promoter primer (5′-TAATACGACTCACTATAGGG) and T7 terminator primer (5′-GCTAGTTATTGCTCAGCGG) in 1x Amplicon *Taq* DNA polymerase master mix (Ampliqon). Sequences were verified by BigDye 3.1 sequencing (Applied Biosystems).

### Recombinant expression

The purified plasmids containing the *bla*_OXA-48_ gene were transformed to *E. coli* strain BL21Star(DE3)pRARE [Invitrogen, Novagen and [25] competent cells by a conventional heat-shock protocol. Cells were used to inoculate 1 L Terrific Broth (TB) media containing 100 μg/ml ampicillin and 34 μg/ml chloramphenicol. Recombinant expression of native OXA-48 was induced by 0.4 mM isopropyl-β-D-thiogalactopyranoside (VWR) at log phase, and expression was continued at 20°C for 16 hours. OXA-48 was isolated from the periplasm using osmotic shock and lysozyme-treatment [26], and purified through two anionic exchange steps, as described previously [27].

### Kinetics

The purified OXA-48 enzyme was used to determine the kinetic parameters for hydrolysis of the substrate meropenem (Sigma) using a Synergy H1 spectrophotometer (BioTek) by measuring the change in absorbance at 300 nm. A plate specific extinction coefficient of 0.002733 OD/μM was used for the UV-plates (Corning). Initial rates were calculated for meropenem concentrations 125-1 μM with 500 nM enzyme in 100 mM Tris-H_2_SO_4_ pH 7, 300 mM Na_2_SO_4_ and 10 mM NaHCO_3_ using Gen5 (BioTek). The steady-state parameters were calculated with non-linear regression using Prism 6 (GraphPad Software).

## Abbreviations

RF: Restriction-free; EMP: Exponential megapriming PCR; OXA: Oxacillinase; ccdB: Coupled cell division B gene; bla: β-Lactamase.

## Competing interests

The authors declare that they have no competing interests.

## Authors’ contributions

BAL carried out the molecular cloning, recombinant expression and kinetics experiments, participated in the design of the study and drafted the manuscript, figure and table. HKSL participated in the design of the study and reviewed the manuscript. GEKB conceived of the study, participated in the design of the study, coordinated and drafted the manuscript and illustration. All authors read and approved the final manuscript.
